# Construction and yield optimization of a cinnamylamine biosynthesis route in *Escherichia coli*

**DOI:** 10.1186/s13068-022-02199-7

**Published:** 2022-09-29

**Authors:** Qi Wang, Linlin Ma, Zhiguo Wang, Quan Chen, Qian Wang, Qingsheng Qi

**Affiliations:** 1 State Key Laboratory of Microbial Technology, National Glycoengineering Research Center, Shandong University, Qingdao, 266237 People’s Republic of China; 2 CAS Key Lab of Biobased Materials, Qingdao Institute of Bioenergy and Bioprocess Technology, Chinese Academy of Sciences, Qingdao, 266101 People’s Republic of China; 3 Institute of Ageing Research, School of Medicine, Hangzhou Normal University, Hangzhou, 311121 People’s Republic of China

**Keywords:** Cinnamylamine biosynthesis, ω-Aminotransferase, Metabolic engineering, Cinnamic acid, *Escherichia coli*

## Abstract

**Background:**

With the development of metabolic engineering and synthetic biology, the biosynthesis of aromatic compounds has attracted much attention. Cinnamylamine is an aromatic compound derived from l-phenylalanine, which is used in the synthesis of biologically active molecules, including drugs, and energetic materials. Cinnamylamine has been mainly synthesized by chemical methods to date, and few reports have focused on the biosynthesis of cinnamylamine. Therefore, it is desirable to establish an efficient biosynthesis method for cinnamylamine.

**Results:**

The ω-aminotransferase Cv-ωTA from *Chromobacterium violaceum* has been demonstrated to have high enzyme activity in the conversion of cinnamaldehyde to cinnamylamine. To prevent the preferable conversion of cinnamaldehyde to cinnamyl alcohol in wild-type *Escherichia coli*, the *E. coli* MG1655 strain with reduced aromatic aldehyde reduction (RARE) in which six aldehyde ketone reductase and alcohol dehydrogenase genes have been knocked out was employed. Then, the carboxylic acid reductase from *Neurospora crassa* (NcCAR) and phosphopantetheinyl transferase (PPTase) from *E. coli* were screened for a high conversion rate of cinnamic acid to cinnamaldehyde. To shift the equilibrium of the reaction toward cinnamylamine, saturation mutagenesis of Cv-ωTA at key amino acid residues was performed, and Cv-ωTA Y168G had the highest conversion rate with 88.56 mg/L cinnamylamine obtained after 4 h of fermentation. Finally, by optimizing the substrates and the supply of the cofactors, PLP and NADPH, in the fermentation, the yield of cinnamylamine in engineered *E. coli* reached 523.15 mg/L.

**Conclusion:**

We achieved the first biosynthesis of cinnamylamine using cinnamic acid as the precursor in *E. coli* using a combinatorial metabolic engineering strategy. This study provides a reference for the biosynthesis of other amine compounds and lays a foundation for the de novo synthesis of cinnamylamine.

**Supplementary Information:**

The online version contains supplementary material available at 10.1186/s13068-022-02199-7.

## Introduction

The microbial production of aromatic compounds is an attractive method because it has the advantages of environmental friendliness, low cost, and feedstock renewability, compared with other synthetic methods [1–6]. Cinnamylamine is an aromatic compound derived from l-phenylalanine (l-Phe). And it is a valuable starting material for synthesizing bioactive substances with antibacterial, antiviral, anticancer, and other pharmacological effects, such as pyrazines and quinazolinones. It is also an essential precursor for energetic compounds that can synthesize energetic materials [7–12]. At present, cinnamylamine is mainly synthesized by chemical methods, and can be obtained by a series of chemical reactions from compounds such as cinnamaldehyde, cinnamyl alcohol or cinnamonitrile as the precursors. The process of synthesizing cinnamylamine by chemical synthesis is relatively mature and has a high conversion rate. But the way to chemically synthesize cinnamylamine usually requires conditions such as high temperature, high pressure and metal catalysts. It has the disadvantages of high energy consumption, environmental pollution and low safety [13–15]. And research on the biocatalysis or microbial biosynthesis of cinnamylamine is limited. Because of the potential applications of cinnamylamine, the development of an environmentally friendly and sustainable method for cinnamylamine biosynthesis is desirable.

The biosynthesis of the direct precursor of cinnamylamine, cinnamaldehyde, from l-Phe has been investigated [2, 16–19]. Cinnamaldehyde has two biosynthetic pathways. In the first, phenylalanine lyase catalyzes the generation of cinnamic acid from phenylalanine that is produced by the shikimate pathway. Then, 4-coumaric acid: CoA ligase (4CL) catalyzes the formation of cinnamoyl-CoA from cinnamic acid, and then cinnamaldehyde is formed from cinnamoyl-CoA under catalysis by cinnamoyl CoA reductase (CCR) [16, 19–25]. In the other pathway, catalysis by carboxylic acid reductase (CAR) and phosphoubiquitin transferase (PPTase) reduces cinnamic acid into cinnamaldehyde [2, 17, 26–32]. To achieve the synthesis of cinnamylamine, it is necessary to obtain an enzyme that catalyzes the generation of cinnamylamine from cinnamaldehyde.

Among the enzymes that convert aldehydes to amines in vitro, transaminase has been shown to be an effective biocatalyst for stereoselective amination. In transaminase catalysis, pyridoxal phosphate (PLP) is usually needed as a cofactor, and the amino group of the amino donor is transferred to an amino acceptor, such as an aldehyde or ketone, to synthesize amine compounds [33–40]. Cerioli et al. have characterized the ω-convertase activity from different sources, *Vibrio fluvialis*, *Chromobacterium violaceum*, and *Halomonas elongata*, using crude cell enzyme solutions [41]. Using different aldehydes, ketones, and alpha-keto acids as substrates, ω-transaminase was demonstrated to be more reactive toward aldehydes and α-keto acids, compared with ketones [41]. In addition, ω-transaminase has been confirmed to recognize and accept cinnamaldehyde as a substrate, which also provides a basis for us to use ω-transaminase to construct the biosynthetic route of cinnamylamine in microorganisms [42, 43]. Thus, it is practicable to achieve biosynthesis and biocatalysis in genetically engineered organisms, such as *E. coli*. In this study, for the first time, a biosynthetic route for cinnamylamine from cinnamic acid was constructed in *E. coli*. By introducing and engineering transaminase Cv-ωTA, blocking the accumulation of the by-product cinnamyl alcohol, screening the pathway enzymes for the conversion of cinnamic acid to cinnamaldehyde, and optimizing the precursors and cofactors, an *E. coli* strain was engineered that had a high rate of cinnamylamine production. Our study expands the available approaches to the biosynthesis of aromatic compounds and provides a reference for the microbial biosynthesis of other amine compounds.

## Results and discussion

### Verification of the catalytic activity of different ω-transaminases toward cinnamaldehyde

To date, the biosynthesis of cinnamylamine has not been achieved, but cinnamaldehyde has been synthetized in *E. coli*, *Saccharomyces cerevisiae*, and *Corynebacterium glutamicum* [16–18]. To accomplish the biosynthesis of cinnamylamine, we investigated the possibility of the conversion of cinnamaldehyde to cinnamylamine. The enzymes that can convert aldehydes to amines in vitro include amine dehydrogenase, monoamine oxidase, and transaminase [44, 45]. Transaminase is often used as a biocatalyst to produce amine compounds [46–49]. Cerioli et al. have shown that ω-transaminase can catalyze the formation of cinnamylamine from cinnamaldehyde in vitro [41]. Therefore, the verification and selection of a highly efficient transaminase is the primary and critical step to accomplish the biosynthesis of cinnamylamine. Two ω-transaminases derived from *C. violaceum* (Cv-ωTA) and *H. elongate* (He-ωTA) were selected as candidates for investigation of the transamination reaction.

**Fig. 1:**
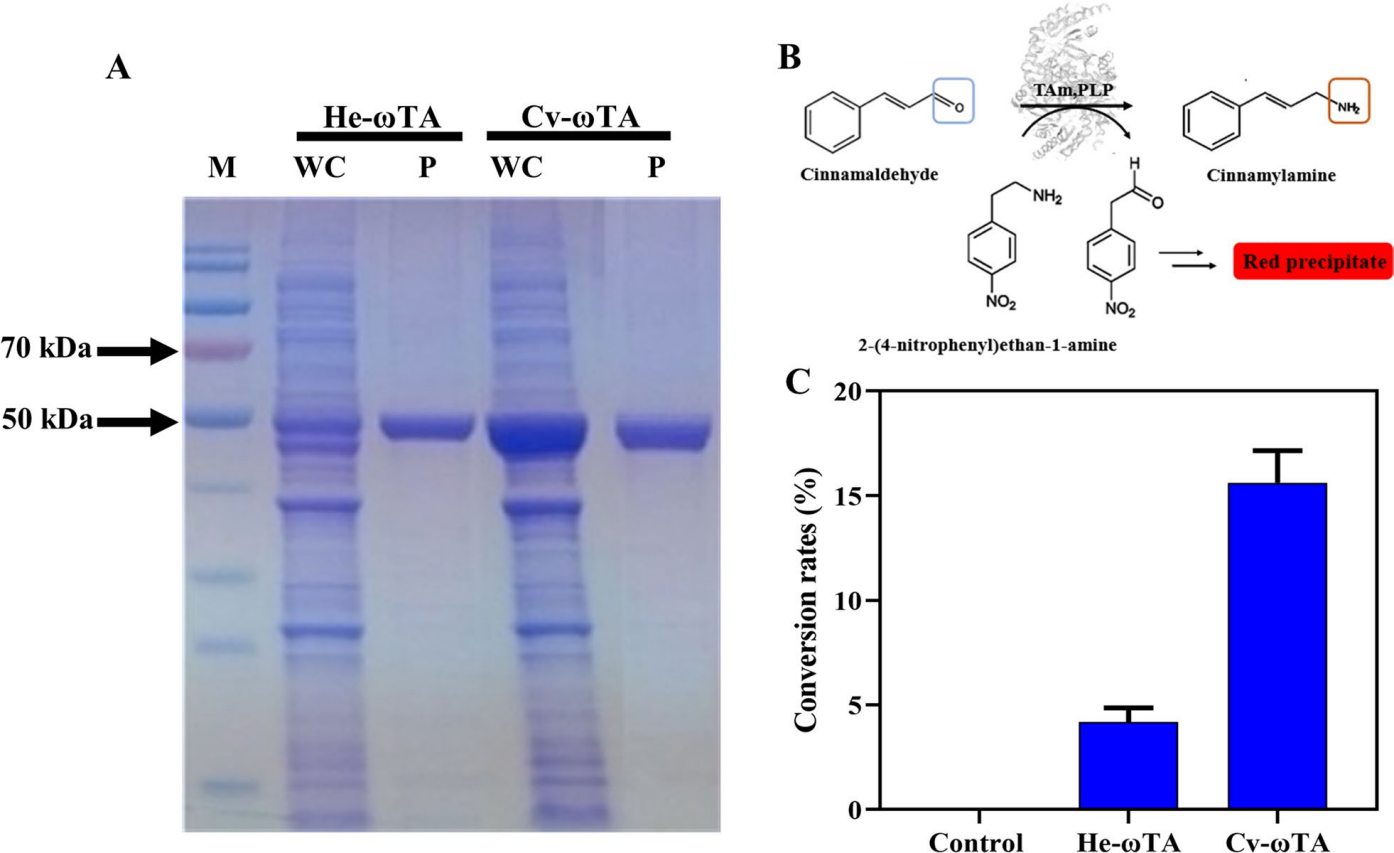
Screening of ω-transaminases from different sources and the determination of the enzymatic activity. **A** He-ωTA (*H. elongate*) and Cv-ωTA (*C. violaceum*) expression by SDS-PAGE analysis of whole cell (WC), purified enzymes (P), and protein marker (M). **B** Validation of the enzymatic activity of ω-transaminase catalyzing the conversion of cinnamaldehyde to cinnamamine. The substrate cinnamaldehyde and 2-(4-nitrophenyl)ethan-1-amine hydrochloride undergo transamination under the action of ω-transaminase to form an imine, and its tautomer produces a red precipitate. The activity of transaminase is reflected by the color change of the chromogenic reaction. **C** Determination of He-ωTA and Cv-ωTA conversion. Buffer without enzyme solution was added as a control. Data represent the mean ± SD (error bars) from three independent experiments

First, the genes coding for Cv-ωTA and He-ωTA were inserted into the backbone of the plasmid pET28a to generate pCT and pHT, respectively, which were subsequently used for the expression and purification of Cv-ωTA and He-ωTA, respectively. The successful expression of the two ω-transaminases in *E. coli* BL21 (DE3) was identified by SDS-PAGE electrophoresis (Fig. 1A). Then, the catalysis activity of toward cinnamaldehyde was identified using a colorimetric method. A red precipitate was formed through the catalysis of the transaminase using 2-(4-nitrophenyl)ethyl-1-amine as the amino donor (Fig. 1B, Additional file 4: Fig. S1). To determine the crude enzyme activity and the conversion rate of the transaminase, l-alanine (l-Ala) was selected as the amino donor, and the conversion rates of cinnamaldehyde to cinnamylamine catalyzed by Cv-ωTA and He-ωTA were 15.62% and 4.18%, respectively (Fig. 1C). Cv-ωTA with a higher conversion rate and activity was selected for further investigation for cinnamylamine biosynthesis.

### Construction of a cinnamylamine biosynthesis pathway using cinnamic acid as the precursor in *E. coli*

**Fig. 2:**
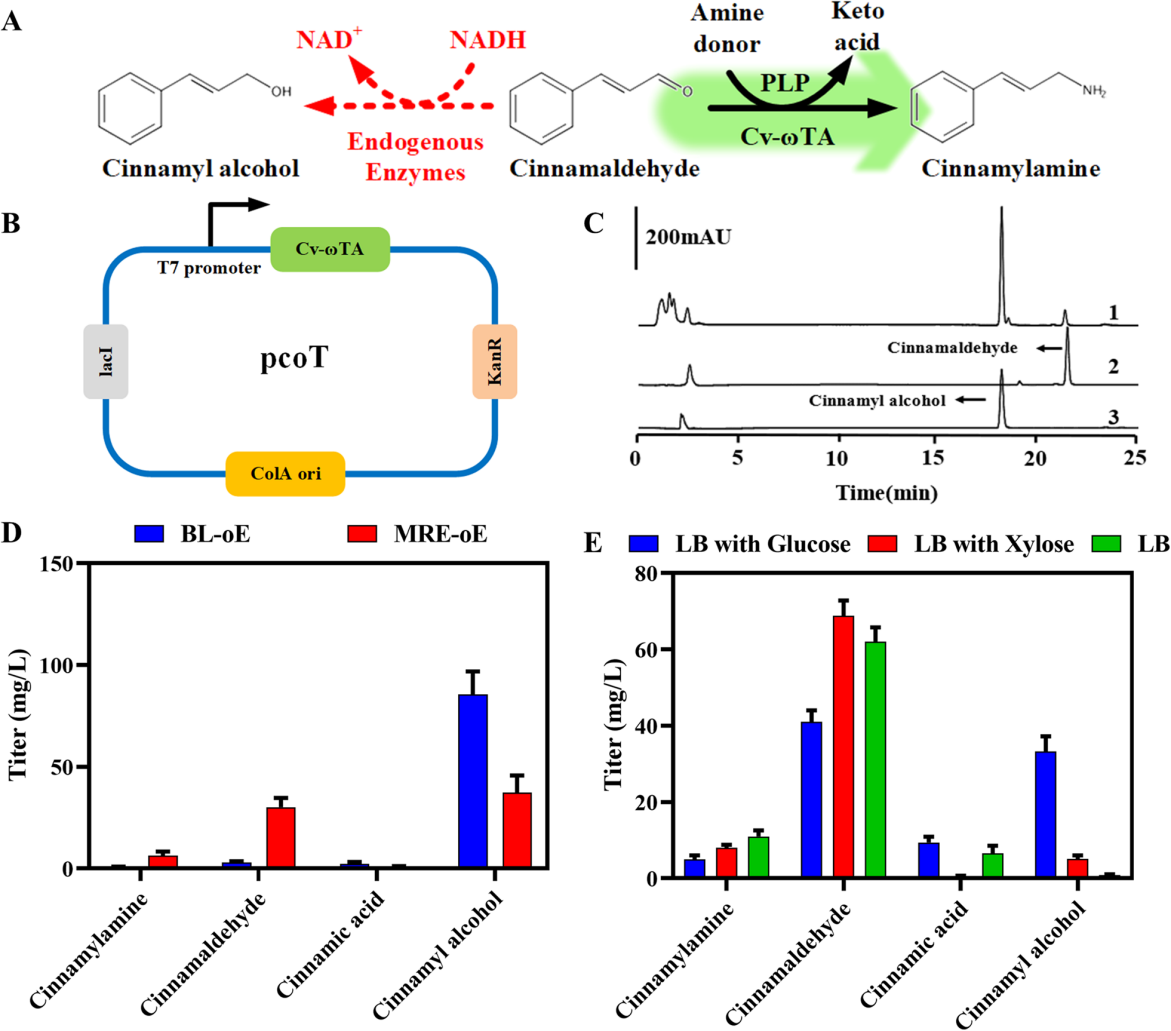
Construction of cinnamylamine biosynthetic pathway using cinnamaldehyde as the precursor in *E. coli*. **A** The conversion pathway of cinnamaldehyde to cinnamyl alcohol and cinnamylamine, and the pcoE plasmid map used to synthesize cinnamylamine using cinnamaldehyde as the precursor. Cv-ωTA: ω-transaminase from *C. violaceum.* Endogenous enzymes: endogenous alcohol dehydrogenase and aldehyde ketone reductase. **B** HPLC chromatograms of cinnamyl alcohol, cinnamaldehyde, and the fermentation broth of the BL-oE strain. 1: HPLC chromatogram of the fermentation broth of the BL-oE strain. 2: HPLC chromatogram of the cinnamaldehyde standard. 3: HPLC chromatogram of the cinnamyl alcohol standard. **C** The accumulation of various substances in the fermentation broth of the BL-oE and MRE-oE strains using cinnamaldehyde as the precursor to produce cinnamylamine. **D** Accumulation of various substances in the fermentation broth of the MRE-oE strain using cinnamaldehyde as the precursor to produce cinnamylamine in media containing different carbon sources. Data represent the mean ± SD (error bars) from three independent experiments

We demonstrated that Cv-ωTA can convert cinnamaldehyde to cinnamylamine in vitro. Next, we examined whether *E. coli* endogenously expressing Cv-ωTA could convert cinnamaldehyde to cinnamylamine. First, we inserted the coding gene for Cv-ωTA into a pCOLADuet plasmid backbone to generate a pcoT plasmid and transformed this plasmid into *E. coli* BL21 (DE3) to obtain the BL-oT strain (Fig. 2B). Then, BL-oE was inoculated into LB medium containing 20 g/L glucose and 100 mg/L cinnamaldehyde and cultured for 4 h for cinnamylamine synthesis. The substrates and products in the fermentation broth were analyzed by HPLC. However, almost no cinnamylamine was detected and, surprisingly, an accumulation of 86.5 mg/L cinnamyl alcohol was present in the cultures (Fig. 2C, D). This large accumulation of cinnamyl alcohol indicated that *E. coli* has abundant and highly active endogenous alcohol dehydrogenase and aldehyde ketone reductase enzymes, which convert cinnamaldehyde to cinnamyl alcohol, and thus restrict the production of cinnamylamine. Kunjapur et al. knocked out three aldehyde ketone reductase genes (*dkgB*, *yeaE*, and *dkgA*) and three alcohol dehydrogenase genes (*yahK*, *yjgB*, and *yqhD*) in *E. coli* MG1655, resulting in the strain MG1655 with reduced aromatic aldehyde reduction (RARE) for aromatic aldehyde production by decreasing the formation of aromatic alcohols (Fig. 2A) [32]. Here, to reduce the synthesis of cinnamyl alcohol, the *E. coli* strain MG1655 (RARE) was used and transformed with pcoT to obtain strain MRE-oE for cinnamylamine production. Using cinnamaldehyde as the precursor, MRE-oE showed a decreased accumulation of cinnamyl alcohol (37.38 mg/L) compared to the BL-oE strain, and an accumulation of 6.45 mg/L cinnamylamine after adding cinnamaldehyde for 4 h (Fig. 2D).

NADH is a required cofactor for the conversion of cinnamaldehyde to cinnamyl alcohol, we believe that the intracellular NADH levels affect the accumulation of cinnamyl alcohol (Fig. 2A). The reducing power provided by different carbon sources is also different, so we optimized the carbon source used in the fermentation medium. Three different fermentation media were compared for cinnamylamine production: LB with 2% glucose; LB with 2% xylose; and LB. Using LB medium supplemented with the precursor cinnamaldehyde for fermentation, the MRE-oE strain produced 11.03 mg/L cinnamylamine with minimal accumulation of cinnamyl alcohol (0.34 mg/L) after 4 h (Fig. 2E). In contrast, the addition of glucose or xylose resulted in less cinnamylamine production and excess cinnamyl alcohol accumulation. In addition, the intracellular content of NADH decreased sequentially in the MRE-oE strains cultured in LB with glucose, LB with xylose, and LB medium, which was consistent with the accumulated concentrations of cinnamyl alcohol (Additional file 5: Fig. S2). Excess glucose or xylose may provide redundant NADH reducing power for the cell, which may have caused the accumulation of cinnamyl alcohol.

**Fig. 3:**
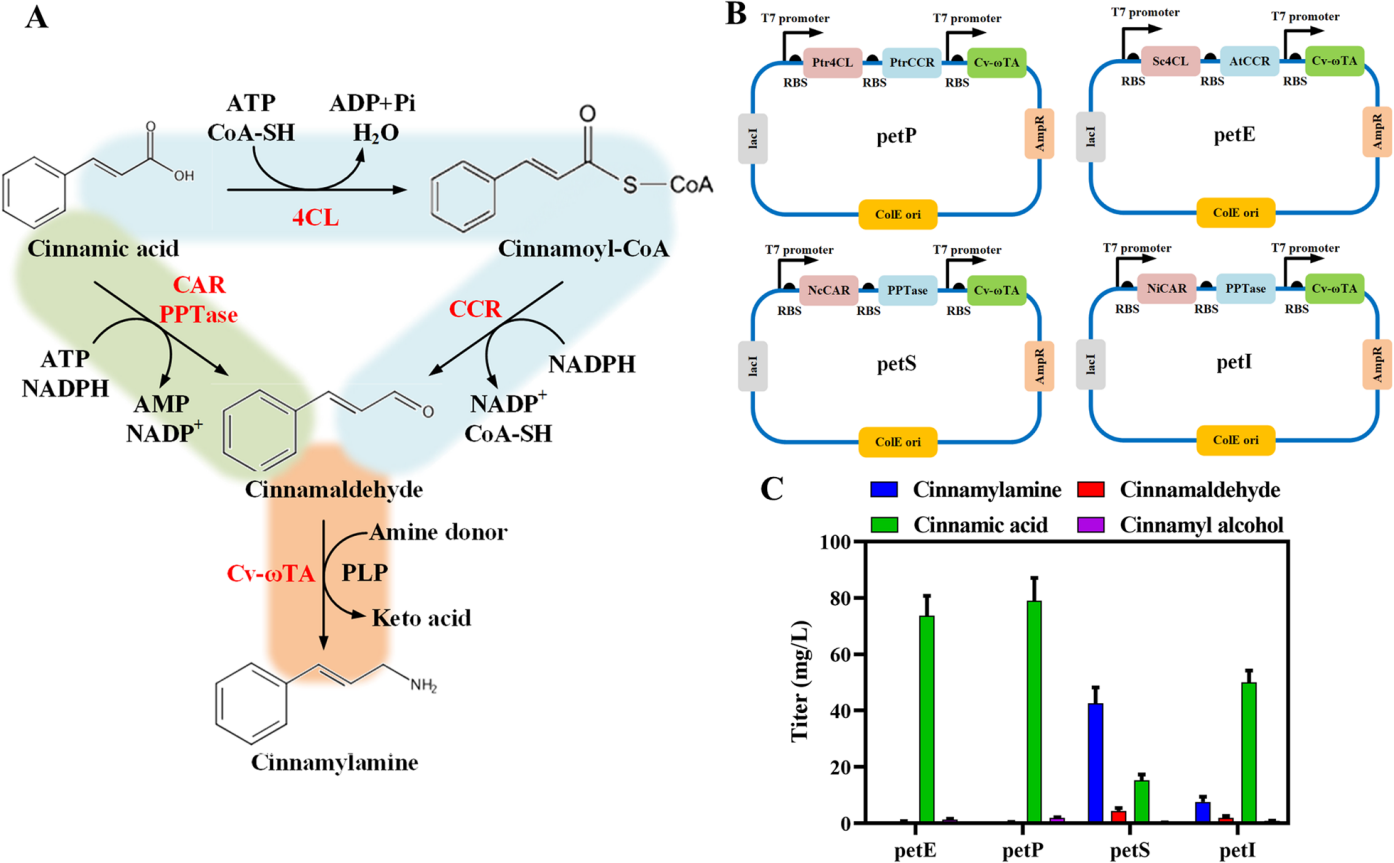
Construction of cinnamylamine biosynthetic pathway using cinnamic acid as the precursor in *E. coli*. **A** The two biosynthetic pathways from cinnamic acid to cinnamylamine and plasmid maps. 4CL: 4-coumarate: CoA ligase. CCR: cinnamyl-CoA reductase. CAR: carboxylic acid reductase. PPTase: phosphopantetheinyl transferase. PtrCCR: cinnamoyl-CoA reductase from *P. trichocarpa.* Ptr4CL: 4-coumarate: CoA ligase from *P. trichocarpa.* Sc4CL: 4-coumarate: CoA ligase from *S. coelicolor.* AtCCR: cinnamoyl-CoA reductase from *A. thaliana.* Cv-ωTA: ω-transaminase from *C. violaceum.* NcCAR: carboxylic acid reductase from *N. crassa*. NiCAR: carboxylic acid reductase from *N. iowensis.* PPTase: phosphopantetheinyl transferase from *E. coli.* **B** Accumulation of various substances in the cinnamylamine production pathway of the MG1655 (RARE) strain using cinnamic acid as the precursor with four different cinnamylamine production plasmids (petP, petE, petS, and petI). Data represent the mean ± SD (error bars) from three independent experiments

To construct a biosynthetic route from cinnamic acid to cinnamylamine in *E. coli*, we screened the pathway enzymes for the conversion of cinnamic acid to cinnamaldehyde. The reduction of cinnamic acid to cinnamaldehyde can be synthesized by a two-step catalytic reaction, through cinnamoyl-CoA, using 4-coumarate: CoA ligase (4CL) and cinnamyl-CoA reductase (CCR), or it can be catalyzed in one step by carboxylic acid reductase (CAR) (Fig. 3A). We selected 4CL from *Streptomyces coelicolor* (Sc4CL) and *Populus trichocarpa* (Ptr4CL); CCR from *Arabidopsis thaliana* (AtCCR) and *P. trichocarpa* (PtrCCR); and CAR from *Neurospora crassa* (NcCAR) and *Nocardia iowensis* (NiCAR) to optimize the cinnamylamine production pathway. The phosphopantetheinyl transferase (PPTase) derived from *E. coli* was expressed together with CAR to ensure the catalytic activity. To improve the expression of pathway enzymes, we used a pETDuet-1 plasmid with a high copy number as the expression vector. Four groups of cinnamaldehyde synthesis enzymes together with Cv-ωTA were cloned into the pETDuet-1 plasmid to obtain four recombinant plasmids petE, petP, petS, and petI (Fig. 3B). Using cinnamic acid as the precursor, the strain MG1655 (RARE) harboring petS produced the highest yield of 42.63 mg/L cinnamylamine after adding cinnamic acid for 4 h (Fig. 3C). Since the cinnamylamine production of the strains harboring the petP or petE plasmids was low, we performed SDS-PAGE analysis of the expression of the enzymes on these two plasmids, and the results showed that the expression of the enzymes on both plasmids is not ideal (Additional file 6: Fig. S3). This may be the reason for its extremely low production of cinnamylamine. But the strains harboring petS or petI plasmid had higher cinnamylamine production. The result showed that the one-step catalysis of cinnamic acid to cinnamaldehyde by NcCAR and PPTase is more beneficial for cinnamylamine production. Therefore, the petS plasmid was used for *E. coli* production of cinnamylamine. We successfully constructed a biosynthetic route for cinnamylamine using cinnamic acid as the precursor in *E. coli.*

### Saturation mutagenesis of Cv-ωTA for improved cinnamylamine production

**Fig. 4:**
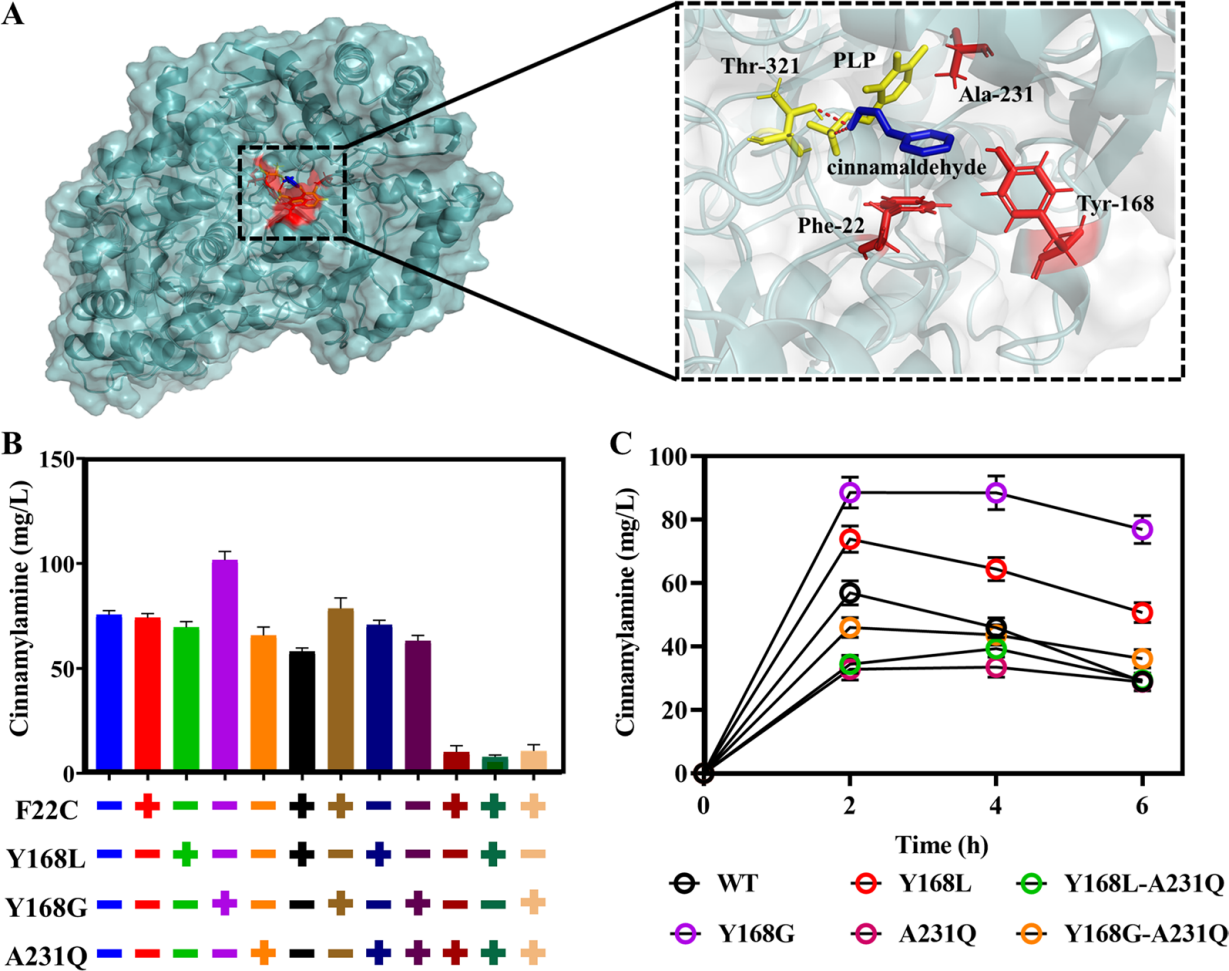
Construction and screening of Cv-ωTA mutants. **A** Key amino acid residues in the substrate-binding pocket of Cv-ωTA (PDB:4AH3). **B** Cinnamylamine production from single-point and combined three amino acid mutants of Cv-ωTA with cinnamaldehyde as the precursor. **C** Cinnamylamine production from enzymatic mutants of Cv-ωTA with cinnamic acid as the precursor. Data represent the mean ± SD (error bars) from three independent experiments

The catalytic reaction of transaminase is balanced between two products. To make the reaction proceed toward cinnamylamine, we designed and modified the transaminase Cv-ωTA to improve the yield of cinnamylamine. Through molecular docking analysis of cinnamaldehyde and Cv-ωTA, three amino acids—Phe22, Tyr168, and Ala231—that are involved in substrate bonding were chosen as the critical sites for saturation mutations (Fig. 4A). Then, a total of 57 Cv-ωTA mutants were obtained using the plasmid pCT and the catalytic efficiency of these mutants was investigated using 200 mg/L cinnamaldehyde as the precursor with cultivation for 4 h. Eight of these mutants gave higher yields and conversion rates for cinnamylamine. Four mutants—F22C (53.64 mg/L), Y168G (70.54 mg/L), Y168L (64.47 mg/L), and A231Q (98.86 mg/L)—had high cinnamylamine conversion rates or yields (Additional file 7: Fig. S4). According to analysis of the molecular docking results for transaminase and cinnamaldehyde, F22C and A231Q may reduce the steric hindrance between the substrate and the surrounding amino acids and thus, are more conducive to substrate binding, compared with the wild-type amino acids. Y168G reduced the steric hindrance and further opened the channel for the substrate to enter the active pocket, making it easier for the substrate to enter and exit, compared with the wild type (Fig. 4A). Subsequently, combinations of the four single-point mutations—F22C, Y168G, Y168L, and A231Q—were investigated to further improve the production of cinnamylamine. Among these mutants, the Y168G mutant had the highest cinnamylamine yield of 101.6 mg/L. Unexpectedly, double and triple mutants did not result in a better yield compared with the single-point mutations. The highest cinnamylamine production of the five double mutants was 78.57 mg/L for the F22C-Y168G mutant. The triple mutants resulted in even lower cinnamylamine yields, and the F22C-Y168G-A231Q mutant gave a cinnamylamine yield of 10.64 mg/L, indicating these combinations of mutations did not have a cumulative effect (Fig. 4B).

On the basis of the above results, we individually replaced the wild-type Cv-ωTA gene in the petS plasmid with five different mutant genes: Y168L, Y168G, A231Q, Y168L-A231Q, and Y168G-A231Q. Then, the cinnamylamine yields were investigated using 200 mg/L cinnamic acid as the precursor. The highest yield of cinnamylamine of 88.56 mg/L was obtained with the Y168G mutant, which was higher than that of the control at 56.95 mg/L (Fig. 4C). Thus, the Cv-ωTA Y168G mutant was selected for subsequent investigation of cinnamylamine production.

### Improvement and optimization of precursors and cofactor supply

**Fig. 5:**
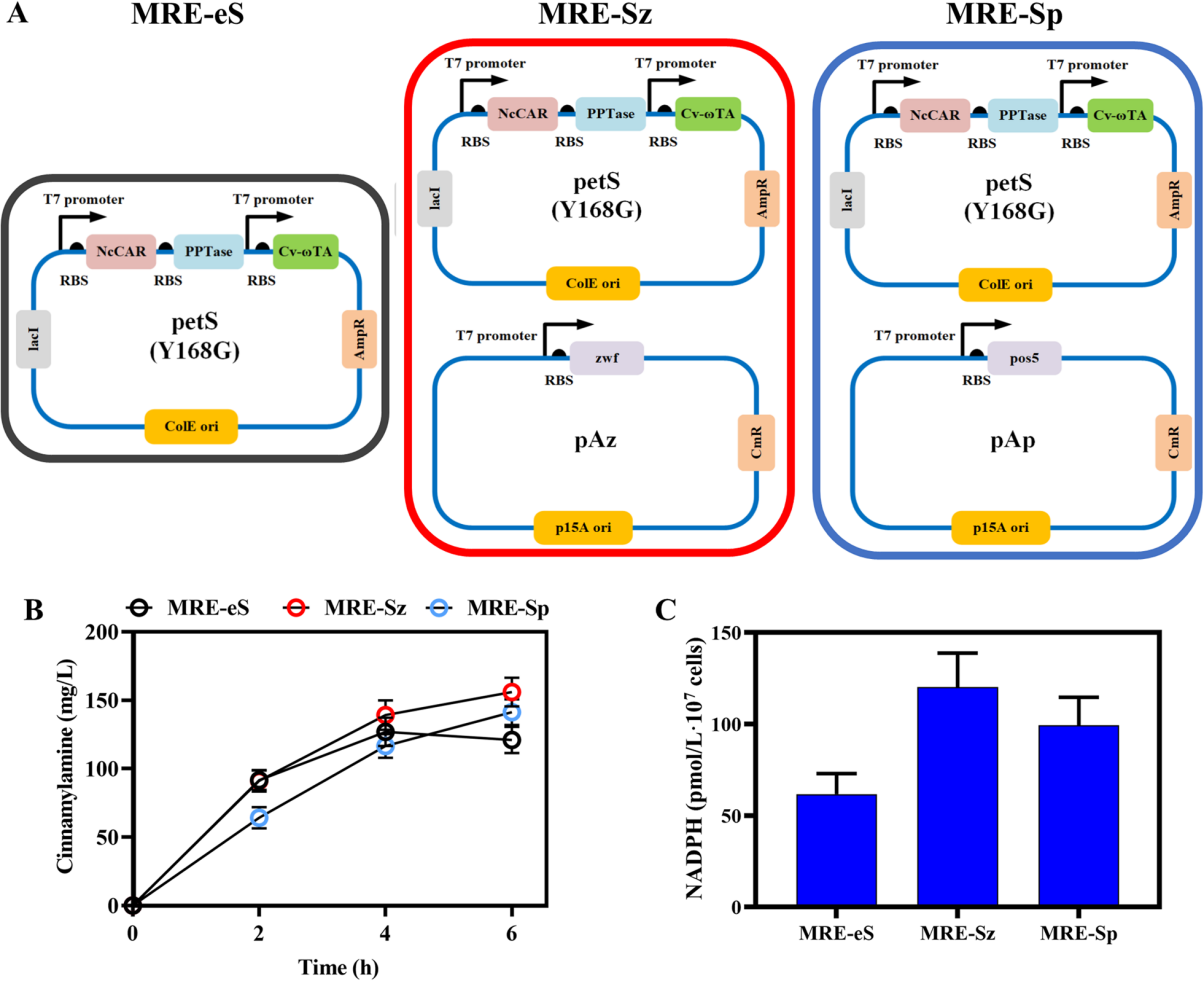
Increased intracellular NADPH cofactor content. **A** The plasmids harboring the three strains: MRE-eS, MRE-Sz, and MRE-Sp. pos5: NAD^+^/NADH kinase. zwf: glucose-6-phosphate dehydrogenase. **B** The biosynthesis of cinnamylamine by the three strains, MRE-eS, MRE-Sz, and MRE-Sp, using cinnamic acid as the precursor. **C** Intracellular NADPH content of MRE-eS, MRE-Sz, and MRE-Sp strains. Data represent the mean ± SD (error bars) from three independent experiments

We successfully improved the catalytic efficiency of transaminase through semi-rational design. However, a large amount of cinnamic acid remained after the fermentation, and the accumulation of cinnamaldehyde was less. We speculated that the conversion of cinnamic acid to cinnamaldehyde was the rate-limiting step. The conversion of cinnamic acid to cinnamaldehyde requires NADPH as a cofactor. Therefore, we investigated increasing the levels of intracellular NADPH to promote the conversion of cinnamic acid to cinnamaldehyde. To increase intracellular NADPH levels, we expressed an NADH kinase that converts NADH to NADPH. The *pos5* gene derived from *S. cerevisiae* was used to convert intracellular NADH to NADPH and the mitochondrial targeting sequence of *pos5* was removed to achieve the soluble expression in recombinant *E. coli*. In addition, glucose-6-phosphate dehydrogenase (encoded by *zwf*) catalyzes the dehydrogenation of glucose-6-phosphate to form 6-phosphogluconic acid and generate NADPH. Thus, the genes *zwf* and *pos5* were inserted into a pACYCduet-1 plasmid to generate pAz and pAp plasmids, respectively. The two plasmids were transformed into the MRE-eS strain MG1655 (RARE) harboring petS(Y168G) to obtain MRE-Sz and MRE-Sp (Fig. 5A). The cinnamylamine production of the MRE-Sz and MRE-Sp strains was 156.38 and 140.73 mg/L, respectively, while the control MRE-eS strain produced 120.06 mg/L (Fig. 5B), after cultivation with 400 mg/L cinnamic acid for 6 h. The intracellular NADPH levels of the three strains indicated that the highest NADPH concentration occurred with the MRE-Sz strain (Fig. 5C), demonstrating that increased NADPH supply is beneficial for cinnamylamine production.

**Fig. 6:**
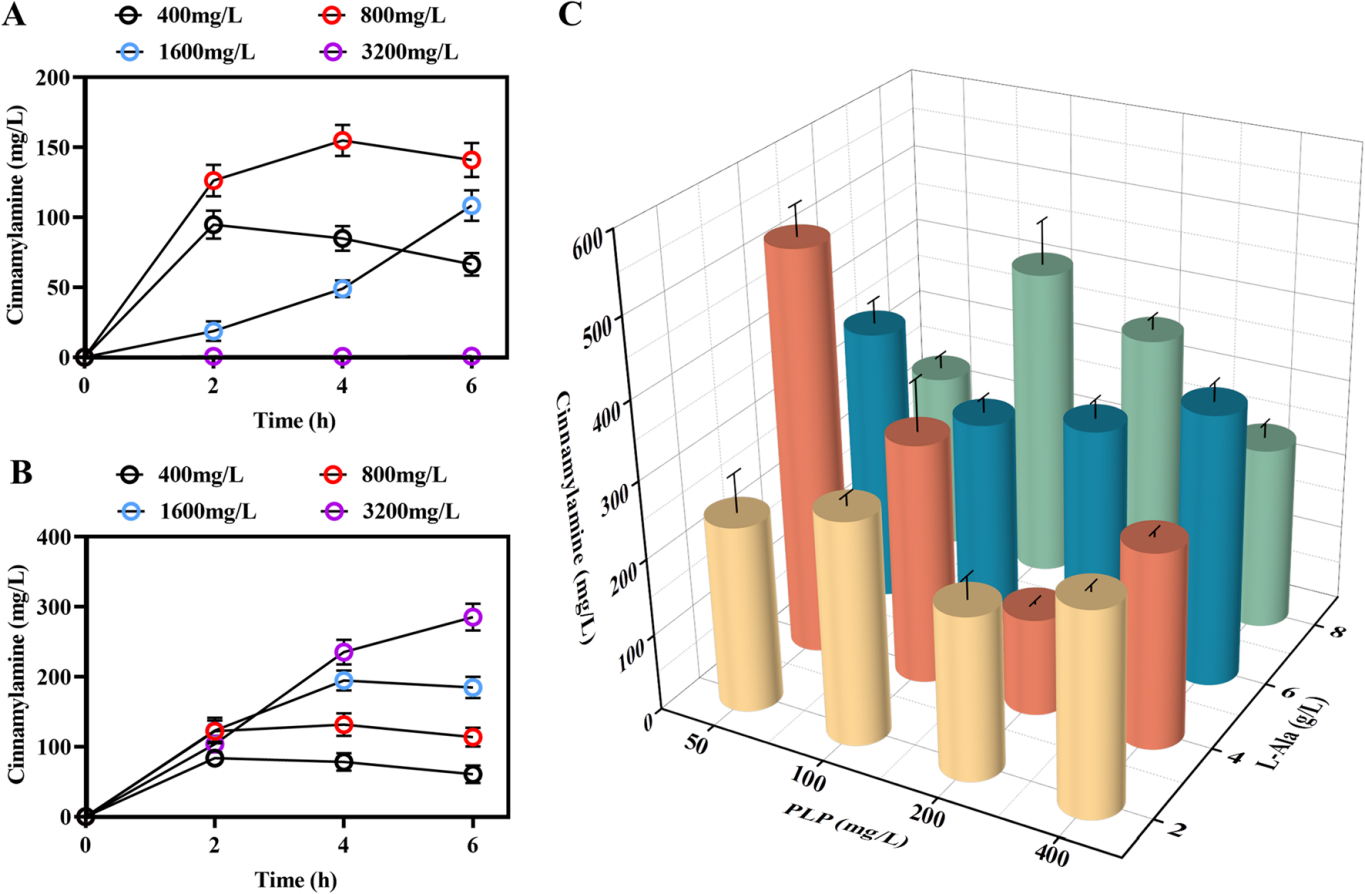
Cinnamylamine yields using the MRE-Sz strain with optimized fermentation conditions. **A** Production of cinnamylamine using the MRE-Sz strain with different concentrations of cinnamic acid as the precursor. **B** Production of cinnamylamine using the MRE-Sz strain with different concentrations of sodium cinnamate as the precursor. **C** The yields of cinnamylamine from the MRE-Sz strain using a combination of different concentrations of PLP and l-Ala. Data represent the mean ± SD (error bars) from three independent experiments

Next, we optimized the fermentation conditions for the production of cinnamylamine. First, we replaced the precursor for cinnamylamine biosynthesis, cinnamic acid, with sodium cinnamate. We found that sodium cinnamate was more favorable for the biosynthesis of cinnamylamine than cinnamic acid (Fig. 6A, B). Therefore, we used 2 g/L sodium cinnamate as the precursor to synthesize cinnamylamine in the subsequent fermentation. We then optimized the concentrations of the amino donor l-alanine (L-Ala) and the cofactor PLP. We used l-alanine concentrations of 2, 4, 6, and 8 g/L as the precursor concentrations. The cofactor PLP was added at concentrations of 50, 100, 200, and 400 mg/L. The different concentrations of l-Ala and PLP were combined in the fermentation. The strain MRE-Sz was fermented using these 16 different combinations. Finally, as shown in the results, using the optimized culture conditions, the yield of cinnamylamine in MRE-Sz reached 523.15 mg/L, using 2 g/L sodium cinnamate, 4 g/L l-alanine, and 50 mg/L PLP, after cultivation for 16 h (Fig. 6C). Therefore, we successfully established a biosynthetic route for cinnamylamine from cinnamic acid in *E. coli*. After our optimization of the cinnamylamine synthesis pathway and culture conditions, the yield of cinnamylamine reached 523.15 mg/L, which provides a foundation for the synthesis of other amine compounds.

To our knowledge, this is the first reported attempt of the biosynthesis of cinnamylamine in *E. coli*. The synthetic route that we constructed is from cinnamic acid to cinnamylamine. To further realize the de novo synthesis of cinnamylamine in *E. coli*, a highly active phenylalanine lyase can be used to catalyze the synthesis of cinnamic acid from phenylalanine.

## Conclusions

In this study, a biosynthetic pathway for the synthesis of cinnamylamine using cinnamic acid as the precursor was successfully constructed in *E. coli*. First, we achieved the biosynthesis of cinnamylamine using cinnamaldehyde as the precursor using a MG1655 (RARE) strain expressing Cv-ωTA. Subsequently, we screened multiple pathway enzymes for the conversion of cinnamic acid to cinnamaldehyde and carried out protein engineering of Cv-ωTA, as well as improving the cofactor supply and optimizing the fermentation conditions. Finally, a biosynthetic pathway for cinnamylamine with cinnamic acid as the precursor was successfully constructed in *E. coli*, and the yield of cinnamylamine reached 523.15 mg/L. This study provides a reference for the biosynthesis of other amine compounds and expands the biosynthesis platform for aromatic compounds. This study also lays the foundation for the de novo synthesis of cinnamylamine.

## Materials and methods

### Bacterial strains and cultivation

The bacterial strains used in this study are listed in Additional file 1: Table S1. E. coli DH5α competent cells were used for plasmid construction. *E. coli* BL21 (DE3) was used for protein expression and purification. *E. coli* was cultivated in a LB (Luria–Bertani) medium at 37 °C and 220 rpm. Appropriate antibiotics were added at the following concentrations: kanamycin (25 μg/mL), ampicillin (50 μg/mL), chloramphenicol (17 μg/mL), and spectinomycin (25 μg/mL).

For cinnamylamine production, 500 μL of seed inoculum from an overnight 5-mL LB culture was added to a 300-mL flask containing 50 mL of LB medium and incubated at 37 °C and 220 rpm. When the OD_600_ value reached 0.6, 0.2 mM IPTG was added for induction. The reagents used were purchased from Sigma (St. Louis, MO, USA).

### Plasmid construction

The constructed plasmids are summarized in Additional file 2: Table S2, and the primers used to construct the plasmids are listed in Additional file 3: Table S3. The target gene and backbone plasmids were amplified by PCR, and the PCR product was purified for plasmid construction. Plasmids were constructed using the ClonExpress II One Step Cloning Kit (Vazyme, Nanjing, China). Cv-ωTA (Uniprot ID: Q7NWG4), He-ωTA (Uniprot ID: E1V913), Sc4CL (Uniprot ID: Q9K3W1), AtCCR (Uniprot ID: Q9S9N9), Ptr4CL (Uniprot ID: B9GXL3), PtrCCR (Uniprot ID: B2Z6Q2), NcCAR (Uniprot ID: Q7RW48), NiCAR (Uniprot ID: Q6RKB1), and pos5 (Uniprot ID: Q06892) were codon optimized and synthesized in *E. coli*. The genes *zwf* (Uniprot ID: P0AC53) and *entD* (Uniprot ID: P19925) were obtained by PCR from the genome of *E. coli* MG1655. The pCT and pHT plasmids were obtained by inserting the coding genes of Cv-ωTA and He-ωTA, respectively, into the pET-28a plasmid backbone with a His tag. The genes encoding Cv-ωTA were inserted into the pCOLA-duet plasmid backbone to generate the pcoT plasmid. The coding genes of the four pairs, Sc4CL and AtCCR; Ptr4CL and PtrCCR; NcCAR and PPTase; and NiCAR and PPTase, and the Cv-ωTA coding gene were inserted into the pETduet-1 plasmid backbone to obtain petE, petP, petS and petI plasmids, respectively. The *pos5* and *zwf* coding genes were inserted into the pACYA-duet-1 plasmid backbone to obtain pAp and pAz plasmids, respectively. Saturation mutagenesis was performed by overlapping PCR using the primers shown in Table S3. All constructed plasmids were verified by colony PCR and Sanger sequencing.

### Purification and enzyme activity assay of transaminase

The BL21 (DE3) strains containing pCT or pHT plasmids were inoculated into 5 mL of LB liquid medium containing kanamycin, and cultured at 37 °C at 220 rpm for 16–18 h, and then were transferred to 500 mL of LB liquid medium containing kanamycin and were incubated for 3 h at 37 °C and 220 rpm until the OD_600_ value reached 0.5–0.7. IPTG was added to induce enzyme expression at 30 °C. After 12 h of culture, the cells were placed in a precooling centrifuge and centrifuged at 8000 rpm for 20 min to harvest the cells. The pellet was resuspended in potassium phosphate buffer containing PLP, adjusted to pH 7.5, and lyophilized. To prepare crude cell extracts, 25 mg of freeze-dried cells was resuspended in potassium phosphate buffer (1 mL, 100 mM, pH 7.5). The resuspended bacterial solution was subjected to ultrasonication to obtain a crude cell extract of the enzyme.

The purification methods of transaminase as previously described [41]. The target protein was eluted from the chromatography column, and the collected fractions for each elution gradient were verified by SDS-PAGE.

When the crude cell extract was tested for enzyme activity, a 24-well plate was used for colorimetric screening. Then, 2-(4-nitrophenyl)ethan-1-amine hydrochloride (25 mM) was added as the amino donor and 10 mM cinnamaldehyde was added as the amino acceptor. PLP (0.2 mM) and potassium phosphate buffer (100 mM) were used to adjust the pH to 7.5. The crude cell extract was added to a 24-well plate and reacted at 37 °C and 500 rpm for 24 h for a color reaction. l-Ala (250 mM, 200 μl), cinnamaldehyde (100 mM, 200 μl), PLP (0.2 mM, 200 μl), and the corresponding enzyme solution were added to the reaction system for enzyme activity assay, and the reaction volume was made up to 1 mL with 100 mM KH_2_PO_4_ buffer. The synthesis of cinnamylamine was detected after 30 min of reaction at 37 °C.

### Molecular docking of Cv-ωTA

The crystal structure of Cv-ωTA has been reported (PDB: 4AH3) [40]. The molecular structure of cinnamaldehyde was downloaded from Pubchem (www.pubchem.org). Cv-ωTA and small molecules were processed using the molecular simulation software Discovery Studio 2017R2. Molecular docking was carried out in accordance with the information of the amino acid residue composition of the protein active site and the substrate-binding pocket that has been reported for Cv-ωTA.

### NADP^+^/NADPH and NAD^+^/NADH quantification assay

To analyze the intracellular NADP^+^/NADPH ratio, *E. coli* were inoculated and cultured in LB medium for 16 h to reach stationary phase, and the cells were harvested by centrifugation (3300×*g*, 4 °C, 10 min). Cells were then washed with ice-cold phosphate-buffered saline, lysed with NADP^+^/NADPH extraction buffer (Biovision, Milpitas, CA, USA) in a microcentrifuge tube, and kept on ice for 10 min. The crude extracts were centrifuged at 15,000×*g* for 10 min to obtain the supernatant. The amount of NADP^+^/NADPH in the cells was measured using a NADP^+^/NADPH Quantitation Colorimetric Kit (Biovision). To measure the intracellular NADH/NAD^+^ ratio, a NAD/NADH-GloTM Assay Kit (Promega, WI, USA) was used. The culture medium was mixed with a DTAB solution for 5 min. To measure NADH, 0.4 N HCl was added to the reaction mixture. Samples were heated to 60 °C for 15 min and cooled to 25 °C before assay, following the manufacturer’s instructions.

### Analytical methods

The quantitative measurement of glucose was performed using an SBA‐40 biosensor analyzer equipped with a glucose oxidase (Institute of Biology of Shandong Province Academy of Sciences, Shandong, China). Optical density (OD) was measured at 600 nm with a spectrophotometer (Shimazu, Japan). The concentrations of cinnamylamine, cinnamaldehyde, cinnamic acid, and cinnamyl alcohol were quantified by HPLC (Shimadzu, Japan) using a C18 reverse phase column (150 mm × 4.6 mm) maintained at 30 °C. The fermentation broth was centrifuged at 12,000 rpm for 2 min, and the supernatant was filtered through a 0.22-μm filter to remove bacteria and impurities for product detection. Trifluoroacetic acid (0.1%; solvent A) and acetonitrile (solvent B) were used as the mobile phases at a flow rate of 0.5 mL min^−1^. The elution was performed in accordance with the following conditions: 0 min 15% B; 16 min 72% B, and 21 min 15% B. The samples were detected at 210 and 250 nm dual wavelengths.

## Supplementary Information

**Additional file 1: Table S1.** Bacterial strains used in this study.

**Additional file 2: Table S2.** Plasmids used in this study.

**Additional file 3: Table S3.** Oligonucleotides used in this study.

**Additional file 4: Figure S1.** Validation of ω-transaminase activity based on color reaction. 1: Control 2: Empty vector plasmid strain breaking solution 3: Breaking solution of strain expressing He-ωTA protein 4: Breaking solution of strain expressing Cv-ωTA protein 5: Purified He-ωTA protein solution 6: Purified Cv-ωTA protein solution.

**Additional file 5: Figure S2.** Intracellular NADH content of MER-oE strains under different medium conditions. Data represent mean ± S.D. (error bars) from three independent experiments.

**Additional file 6: Figure S3.** The petP and petE plasmids expression in *E. coil* by SDS-PAGE analysis of whole cell (WC), supernatant (S), and protein marker (M).

**Additional file 7: Figure S4.** Yields of saturated mutants of three amino acid residues of Cv-ωTA converting cinnamaldehyde to cinnamylamine. (A) The production of cinnamylamine in saturated mutants of at the Phe22 site after adding cinnamaldehyde. (B) The production of cinnamylamine in saturated mutants of at the Tyr168 site after adding cinnamaldehyde. (C) The production of cinnamylamine in saturated mutants of at the Ala231 site after adding cinnamaldehyde. The mutant with the highest cinnamylamine production among the saturated mutants at each site is marked with a red inverted triangle. "X" represents any amino acid. Data represent mean ± S.D. (error bars) from three independent experiments.

## Data Availability

All data generated or analyzed during this study are included in this published article and its additional materials.
