# Supplementary material for: Construction and yield optimization of a cinnamylamine biosynthesis route in Escherichia coli
Source: Biotechnol Biofuels Bioprod. 2022 Sep 29;15:100. doi: 10.1186/s13068-022-02199-7 (PMC9524069; doi:10.1186/s13068-022-02199-7)
Supplement: Supplementary file 1 — Additional file 1: Table S1. Bacterial strains used in this study. [file 13068_2022_2199_MOESM1_ESM.docx]

**Table S1. Bacterial strains used in this study**

| **Strains** | **Relevant characteristics** | **Sources** |
| --- | --- | --- |
| *E.coli* BL21 (DE3) | F-*omp*T *hsd*SB (rB-mB-) *gal dcm* (DE3) | Lab stock |
| *E.coli* MG1655 (RARE) | *E.coli* MG1655 derivative ∆*dkgB∆yeaE∆dkgA∆yahK∆yjgB∆yqhD* | (1) |
| MRE-tE | MG1655 (RARE) harboring petE | This study |
| MRE-oE | MG1655 (RARE) harboring pcoT | This study |
| BL-oE | *E. coli* BL21 (DE3) harboring pcoT | This study |
| MRE- CT  BL- CE  BL-HT  MRE-HT | MG1655 (RARE) harboring pCT  *E. coli* BL21 (DE3) harboring pCT  *E. coli* BL21 (DE3) harboring pHT  MG1655 (RARE) harboring pHT | This study  This study  This study  This study |
| MRE-tP | MG1655 (RARE) harboring petP | This study |
| MRE-tS | MG1655 (RARE) harboring petS | This study |
| MRE-tI  MRE-eS  MRS-L  MRS-Q  MRS-C  MRS-CL  MRS-CG  MRS-GQ  MRS-LQ  MRS-CQ  MRS-CLQ  MRS-CGQ  MRE- Sz  MRE- Sp | MG1655 (RARE) harboring petI  MG1655 (RARE) harboring MU-G  MG1655 (RARE) harboring Mu-L  MG1655 (RARE) harboring Mu-Q  MG1655 (RARE) harboring Mu-C  MG1655 (RARE) harboring Mu-CL  MG1655 (RARE) harboring Mu-CG  MG1655 (RARE) harboring Mu-GQ  MG1655 (RARE) harboring Mu-LQ  MG1655 (RARE) harboring Mu-CQ  MG1655 (RARE) harboring Mu-CLQ  MG1655 (RARE) harboring Mu-CGQ  MRE-eS harboring pAz  MRE-eS harboring pAp | This study  This study  This study  This study  This study  This study  This study  This study  This study  This study  This study  This study  This study  This study |

**References**

1. Kunjapur AM, Tarasova Y, Prather KL. Synthesis and accumulation of aromatic aldehydes in an engineered strain of Escherichia coli. J Am Chem Soc. 2014;136(33):11644-54.
