# Supplementary material for: Construction and yield optimization of a cinnamylamine biosynthesis route in Escherichia coli
Source: Biotechnol Biofuels Bioprod. 2022 Sep 29;15:100. doi: 10.1186/s13068-022-02199-7 (PMC9524069; doi:10.1186/s13068-022-02199-7)
Supplement: Supplementary file 2 — Additional file 2: Table S2. Plasmids used in this study. [file 13068_2022_2199_MOESM2_ESM.docx]

**Table S2. Plasmids used in this study**

| **Plasmids** | **Relevant characteristics** | **Sources** |
| --- | --- | --- |
| pET-28a | Expression vector, *Kan^R^* | Lab stock |
| pColADuet-1 | Expression vector,*Kan^R^* | Lab stock |
| pACYCDuet-1 | Expression vector, *Cm^R^* | Lab stock |
| pETDuet-1 | Expression vector,*Amp^R^* | Lab stock |
| pCT | pET-28a with Cv-ωTA coding sequence | This study |
| pHT | pET-28a with He-TA coding sequence | This study |
| pcoT | pColADuet-1 with Cv-ωTA coding sequence | This study |
| petE | pETDuet-1 with Sc4CL, AtCCR, Cv-ωTA coding sequence | This study |
| petS | pETDuet-1 with NcCAR, PPTase, Cv-ωTA coding sequence | This study |
| petI | pETDuet-1 with NiCAR, PPTase, Cv-ωTA coding sequence | This study |
| petP | pETDuet-1 with Ptr4CL, PtrCCR, Cv-ωTA coding sequence | This study |
| pAz | pACYC-Duet-1 with *zwf* | This study |
| PAp | pACYC-Duet-1 with *pos5* | This study |
| Mu-C | petS with F22C mutant transaminase sequence | This study |
| Mu-G | petS with Y168G mutant transaminase sequence | This study |
| Mu-L | petS with Y168L mutant transaminase sequence | This study |
| Mu-Q | petS with A231Q mutant transaminase sequence | This study |
| Mu-LQ | petS with Y168L and A231Q mutant transaminase sequence | This study |
| Mu-GQ | petS with Y168G and A231Q mutant transaminase sequence | This study |
| Mu-CQ | petS with F22C and A231Q mutant transaminase sequence | This study |
| Mu-CLQ | petS with F22C、Y168L and A231Q mutant transaminase sequence | This study |
| Mu-CGQ | petS with F22C、Y168G and A231Q mutant transaminase sequence | This study |
