# Supplementary material for: Construction and yield optimization of a cinnamylamine biosynthesis route in Escherichia coli
Source: Biotechnol Biofuels Bioprod. 2022 Sep 29;15:100. doi: 10.1186/s13068-022-02199-7 (PMC9524069; doi:10.1186/s13068-022-02199-7)
Supplement: Supplementary file 3 — Additional file 3: Table S3. Oligonucleotides used in this study. [file 13068_2022_2199_MOESM3_ESM.docx]

**Table S3. Oligonucleotides used in this study**

| **Primer name** | **Sequence** |
| --- | --- |
| pcoECCL-F | TACCATGGGCAGCAGCCATCACCATCATCACCACTTCCGTTCTGAATACGCTGACGTT |
| pcoECCL-R1 | AACAAAATTATTTCTACAGGTCGGATCCTGGCTTAACGCGGTTCACGCAGCTGA |
| pcoECCL-R2 | ATATCTCCTTATTAAAGTTAAACAAAATTATTTCTACAGGTCGGATCCTGG |
| pcoECCR-F | AATAATTTTGTTTAACTTTAATAAGGAGATATACCATGCTGGTTGACGGTAAACTGGTT |
| pcoECCR-R | TCGACTTAAGCATTATGCGGCCGCTTAAGATTCGATGATAACTTCGTTCTGGTTAGAGT |
| pcoETA-F | TTAGTTAAGTATAAGAAGGAGATATACATATGCAGAAACAGCGCACCACCAGT |
| pcoETA-R | TCGCAGCAGCGGTTTCTTTACCAGACTCGAGTTAGGCCAGGCCACGTGCTTT |
| pcoE-ccGJ-F | GATGATGGTGATGGCTGCTGCCCA |
| pcoE-ccGJ-R | TTATCATCGAATCTTAAGCGGCCGCATAATGCTTAAGTCG |
| pcoETA-cctGJ-F | TCGAGTCTGGTAAAGAAACCGCTGCTG |
| pcoETA-cctGJ-R | CATATGTATATCTCCTTCTTATACTTAACTAA |
| pcoE -test-F | CCGGCATACTCTGCGACATCGTATA |
| pcoE -test-R | CAGGATTTCCTGAACACCACCAG |
| Copy-F | ATAAGGAGATATACCATGGGCAGCAG |
| Copy-R | CTTTACCAGACTCTCGAGTTAGGCCAGGCCACG |
| Copy-GJ-F | TTGTACACGGCCGCATAATC |
| Copy-GJ-R | ATGGCTGCTGCCCATGGTATATC |
| Copy-test-F | GTGAGCGGATAACAATTCCCCTG |
| Copy-acE-test-R | TTGCTGGTTTACCGGTTTATTG |
| Copy-cdE-test-R | TTGCTGGTTTACCGGTTTATTG |
| Copy-etE-test-R | GCTAGTTATTGCTCAGCGG |
| pCT-F | TCTAGAAATAATTTTGTTTAACTTTAAGAAGGAGATATACATATGCAGAAGCAACGTACGAC |
| pCT-R | GTGGTGGTGGTGGTGCTCGAGCTAAGCCAGCCCGCGCGCCTT |
| pCT-GJ-F | CGCGCGGGCTGGCTTAGCTCGAGCACCACCACCACCAC |
| pCT-GJ-R | CTTCTTAAAGTTAAACAAAATTATTTCTAGAGGGGAATTGTTATCC |
| NC-R | AAAATTATTTCTACAGGTCGGATCCTGGCTTAAAAGTTCCACTGACGACACCAATTTTT |
| PT-F | AATAAGGAGATATACCatgaaaactacgcatacctcc |
| PT-R | CTGTTCGACTTAAGCATTATGCGGCCGCTttaatcgtgttggcacagcg |
| NP-GJ-F | ATGGTGATGGCTGCTGCCCA |
| NP-GJ-R | GATTAAGCGGCCGCATAATGCTTAAG |
| NP-test-F | tgataagagacaccggcatactctgc |
| NP-test-R | TTTCGGGGTGCCGGTGCTA |
| 22-A-F | TCATCTGCATCCGGCAACCGATACCGCAAGTCTGAATCAGGCAGGCGCCC |
| 22-A-F | CTTGCGGTATCGGTTGCCGGATGCAGATGATGGGCGGCATCCAGTTCACG |
| 22-R-F | TCATCTGCATCCGCGCACCGATACCGCAAGTCTGAATCAGGCAGGCGCCC |
| 22-R-R | CTTGCGGTATCGGTGCGCGGATGCAGATGATGGGCGGCATCCAGTTCACG |
| 22-N-F | TCATCTGCATCCGAATACCGATACCGCAAGTCTGAATCAGGCAGGCGCCC |
| 22-N-R | CTTGCGGTATCGGTATTCGGATGCAGATGATGGGCGGCATCCAGTTCACG |
| 22-D-F | TCATCTGCATCCGGATACCGATACCGCAAGTCTGAATCAGGCAGGCGCCC |
| 22-D-R | CTTGCGGTATCGGTATCCGGATGCAGATGATGGGCGGCATCCAGTTCACG |
| 22-C-F | TCATCTGCATCCGTGTACCGATACCGCAAGTCTGAATCAGGCAGGCGCCC |
| 22-C-R | CTTGCGGTATCGGTACACGGATGCAGATGATGGGCGGCATCCAGTTCACG |
| 22-Q-F | TCATCTGCATCCGCAGACCGATACCGCAAGTCTGAATCAGGCAGGCGCCC |
| 22-Q-R | CTTGCGGTATCGGTCTGCGGATGCAGATGATGGGCGGCATCCAGTTCACG |
| 22-E-F | TCATCTGCATCCGGAAACCGATACCGCAAGTCTGAATCAGGCAGGCGCCC |
| 22-E-R | CTTGCGGTATCGGTTTCCGGATGCAGATGATGGGCGGCATCCAGTTCACG |
| 22-G-F | TCATCTGCATCCGGGTACCGATACCGCAAGTCTGAATCAGGCAGGCGCCC |
| 22-G-R | CTTGCGGTATCGGTACCCGGATGCAGATGATGGGCGGCATCCAGTTCACG |
| 22-H-F | TCATCTGCATCCGCATACCGATACCGCAAGTCTGAATCAGGCAGGCGCCC |
| 22-H-R | CTTGCGGTATCGGTATGCGGATGCAGATGATGGGCGGCATCCAGTTCACG |
| 22-I-F | TCATCTGCATCCGATTACCGATACCGCAAGTCTGAATCAGGCAGGCGCCC |
| 22-I-R | CTTGCGGTATCGGTAATCGGATGCAGATGATGGGCGGCATCCAGTTCACG |
| 22-L-F | TCATCTGCATCCGCTGACCGATACCGCAAGTCTGAATCAGGCAGGCGCCC |
| 22-L-R | CTTGCGGTATCGGTCAGCGGATGCAGATGATGGGCGGCATCCAGTTCACG |
| 22-K-F | TCATCTGCATCCGAAAACCGATACCGCAAGTCTGAATCAGGCAGGCGCCC |
| 22-K-R | CTTGCGGTATCGGTTTTCGGATGCAGATGATGGGCGGCATCCAGTTCACG |
| 22-M-F | TCATCTGCATCCGATGACCGATACCGCAAGTCTGAATCAGGCAGGCGCCC |
| 22-M-R | CTTGCGGTATCGGTCATCGGATGCAGATGATGGGCGGCATCCAGTTCACG |
| 22-P-F | TCATCTGCATCCGCCGACCGATACCGCAAGTCTGAATCAGGCAGGCGCCC |
| 22-P-R | CTTGCGGTATCGGTCGGCGGATGCAGATGATGGGCGGCATCCAGTTCACG |
| 22-S-F | TCATCTGCATCCGAGTACCGATACCGCAAGTCTGAATCAGGCAGGCGCCC |
| 22-S-R | CTTGCGGTATCGGTACTCGGATGCAGATGATGGGCGGCATCCAGTTCACG |
| 22-T-F | TCATCTGCATCCGACCACCGATACCGCAAGTCTGAATCAGGCAGGCGCCC |
| 22-T-R | CTTGCGGTATCGGTGGTCGGATGCAGATGATGGGCGGCATCCAGTTCACG |
| 22-W-F | TCATCTGCATCCGTGGACCGATACCGCAAGTCTGAATCAGGCAGGCGCCC |
| 22-W-R | CTTGCGGTATCGGTCCACGGATGCAGATGATGGGCGGCATCCAGTTCACG |
| 22-Y-F | TCATCTGCATCCGTATACCGATACCGCAAGTCTGAATCAGGCAGGCGCCC |
| 22-Y-R | CTTGCGGTATCGGTATACGGATGCAGATGATGGGCGGCATCCAGTTCACG |
| 22-V-F | TCATCTGCATCCGGTTACCGATACCGCAAGTCTGAATCAGGCAGGCGCCC |
| 22-V-R | CTTGCGGTATCGGTAACCGGATGCAGATGATGGGCGGCATCCAGTTCACG |
| 22-test-F | GACGCTCTCCCTTATGCGAC |
| 22-test-R | CCCAATAACGACGCACCATACG |
| 168-A-F | AAGCCTGGGCGGCATGAAAGCCATGCATGAACAGGGCGATCTGCCGATTC |
| 168-A-F | TCATGCATGGCTTTCATGCCGCCCAGGCTTGCGCCGCCAATGGTACTACC |
| 168-R-F | AAGCCTGGGCGGCATGAAACGCATGCATGAACAGGGCGATCTGCCGATTC |
| 168-R-R | TCATGCATGCGTTTCATGCCGCCCAGGCTTGCGCCGCCAATGGTACTACC |
| 168-N-F | AAGCCTGGGCGGCATGAAAAATATGCATGAACAGGGCGATCTGCCGATTC |
| 168N-R | TCATGCATATTTTTCATGCCGCCCAGGCTTGCGCCGCCAATGGTACTACC |
| 168-D-F | AAGCCTGGGCGGCATGAAAGATATGCATGAACAGGGCGATCTGCCGATTC |
| 168-D-R | TCATGCATATCTTTCATGCCGCCCAGGCTTGCGCCGCCAATGGTACTACC |
| 168-C-F | AAGCCTGGGCGGCATGAAATGCATGCATGAACAGGGCGATCTGCCGATTC |
| 168-C-R | TCATGCATGCATTTCATGCCGCCCAGGCTTGCGCCGCCAATGGTACTACC |
| 168-Q-F | AAGCCTGGGCGGCATGAAACAGATGCATGAACAGGGCGATCTGCCGATTC |
| 168-Q-R | TCATGCATCTGTTTCATGCCGCCCAGGCTTGCGCCGCCAATGGTACTACC |
| 168-E-F | AAGCCTGGGCGGCATGAAAGAAATGCATGAACAGGGCGATCTGCCGATTC |
| 168-E-R | TCATGCATTTCTTTCATGCCGCCCAGGCTTGCGCCGCCAATGGTACTACC |
| 168-G-F | AAGCCTGGGCGGCATGAAAGGCATGCATGAACAGGGCGATCTGCCGATTC |
| 168-G-R | TCATGCATGCCTTTCATGCCGCCCAGGCTTGCGCCGCCAATGGTACTACC |
| 168-H-F | AAGCCTGGGCGGCATGAAACATATGCATGAACAGGGCGATCTGCCGATTC |
| 168-H-R | TCATGCATATGTTTCATGCCGCCCAGGCTTGCGCCGCCAATGGTACTACC |
| 168-I-F | AAGCCTGGGCGGCATGAAAATTATGCATGAACAGGGCGATCTGCCGATTC |
| 168-I-R | TCATGCATAATTTTCATGCCGCCCAGGCTTGCGCCGCCAATGGTACTACC |
| 168-L-F | AAGCCTGGGCGGCATGAAACTGATGCATGAACAGGGCGATCTGCCGATTC |
| 168-L-R | TCATGCATCAGTTTCATGCCGCCCAGGCTTGCGCCGCCAATGGTACTACC |
| 168-K-F | AAGCCTGGGCGGCATGAAAAAAATGCATGAACAGGGCGATCTGCCGATTC |
| 168-K-R | TCATGCATTTTTTTCATGCCGCCCAGGCTTGCGCCGCCAATGGTACTACC |
| 168-M-F | AAGCCTGGGCGGCATGAAAATGATGCATGAACAGGGCGATCTGCCGATTC |
| 168-M-R | TCATGCATCATTTTCATGCCGCCCAGGCTTGCGCCGCCAATGGTACTACC |
| 168-P-F | AAGCCTGGGCGGCATGAAACCGATGCATGAACAGGGCGATCTGCCGATTC |
| 168-P-R | TCATGCATCGGTTTCATGCCGCCCAGGCTTGCGCCGCCAATGGTACTACC |
| 168-S-F | AAGCCTGGGCGGCATGAAAAGTATGCATGAACAGGGCGATCTGCCGATTC |
| 168-S-R | TCATGCATACTTTTCATGCCGCCCAGGCTTGCGCCGCCAATGGTACTACC |
| 168-T-F | AAGCCTGGGCGGCATGAAAACCATGCATGAACAGGGCGATCTGCCGATTC |
| 168-T-R | TCATGCATGGTTTTCATGCCGCCCAGGCTTGCGCCGCCAATGGTACTACC |
| 168-F-F | AAGCCTGGGCGGCATGAAATTTATGCATGAACAGGGCGATCTGCCGATTC |
| 168-F-R | TCATGCATAAATTTCATGCCGCCCAGGCTTGCGCCGCCAATGGTACTACC |
| 168-W-F | AAGCCTGGGCGGCATGAAATGGATGCATGAACAGGGCGATCTGCCGATTC |
| 168-W-R | TCATGCATCCATTTCATGCCGCCCAGGCTTGCGCCGCCAATGGTACTACC |
| 168-V-F | AAGCCTGGGCGGCATGAAAGTGATGCATGAACAGGGCGATCTGCCGATTC |
| 168-V-R | TCATGCATCCATTTCATGCCGCCCAGGCTTGCGCCGCCAATGGTACTACC |
| 168-test-F | CGCAAGGAATGGTGCATGCA |
| 168-test-R | GCGGTAAACAGATCCGGCTG |
| 231-R-F | GAACCGATTCAGGGTCGTGGTGGCGTTATTGTTCCGCCGGCCACCTATTG |
| 231-R-R | AATAACGCCACCACGACCCTGAATCGGTTCGCCAACAAATGCTGCCACTT |
| 231-N-F | GAACCGATTCAGGGTAATGGTGGCGTTATTGTTCCGCCGGCCACCTATTG |
| 231-N-R | AATAACGCCACCATTACCCTGAATCGGTTCGCCAACAAATGCTGCCACTT |
| 231-D-F | GAACCGATTCAGGGTGATGGTGGCGTTATTGTTCCGCCGGCCACCTATTG |
| 231-D-R | AATAACGCCACCATCACCCTGAATCGGTTCGCCAACAAATGCTGCCACTT |
| 231-C-F | GAACCGATTCAGGGTTGCGGTGGCGTTATTGTTCCGCCGGCCACCTATTG |
| 231-C-R | AATAACGCCACCGCAACCCTGAATCGGTTCGCCAACAAATGCTGCCACTT |
| 231-Q-F | GAACCGATTCAGGGTCAGGGTGGCGTTATTGTTCCGCCGGCCACCTATTG |
| 231-Q-R | AATAACGCCACCCTGACCCTGAATCGGTTCGCCAACAAATGCTGCCACTT |
| 231-E-F | GAACCGATTCAGGGTGAAGGTGGCGTTATTGTTCCGCCGGCCACCTATTG |
| 231-E-R | AATAACGCCACCTTCACCCTGAATCGGTTCGCCAACAAATGCTGCCACTT |
| 231-G-F | GAACCGATTCAGGGTGGCGGTGGCGTTATTGTTCCGCCGGCCACCTATTG |
| 231-G-R | AATAACGCCACCGCCACCCTGAATCGGTTCGCCAACAAATGCTGCCACTT |
| 231-H-F | GAACCGATTCAGGGTCATGGTGGCGTTATTGTTCCGCCGGCCACCTATTG |
| 231-H-R | AATAACGCCACCATGACCCTGAATCGGTTCGCCAACAAATGCTGCCACTT |
| 231-I-F | GAACCGATTCAGGGTATTGGTGGCGTTATTGTTCCGCCGGCCACCTATTG |
| 231-I-R | AATAACGCCACCAATACCCTGAATCGGTTCGCCAACAAATGCTGCCACTT |
| 231-L-F | GAACCGATTCAGGGTTTGGGTGGCGTTATTGTTCCGCCGGCCACCTATTG |
| 231-L-R | AATAACGCCACCCAAACCCTGAATCGGTTCGCCAACAAATGCTGCCACTT |
| 231-K-F | GAACCGATTCAGGGTAAAGGTGGCGTTATTGTTCCGCCGGCCACCTATTG |
| 231-K-R | AATAACGCCACCTTTACCCTGAATCGGTTCGCCAACAAATGCTGCCACTT |
| 231-M-F | GAACCGATTCAGGGTATGGGTGGCGTTATTGTTCCGCCGGCCACCTATTG |
| 231-M-R | AATAACGCCACCCATACCCTGAATCGGTTCGCCAACAAATGCTGCCACTT |
| 231-P-F | GAACCGATTCAGGGTCCAGGTGGCGTTATTGTTCCGCCGGCCACCTATTG |
| 231-P-R | AATAACGCCACCTGGACCCTGAATCGGTTCGCCAACAAATGCTGCCACTT |
| 231-S-F | GAACCGATTCAGGGTAGCGGTGGCGTTATTGTTCCGCCGGCCACCTATTG |
| 231-S-R | AATAACGCCACCGCTACCCTGAATCGGTTCGCCAACAAATGCTGCCACTT |
| 231-T-F | GAACCGATTCAGGGTACCGGTGGCGTTATTGTTCCGCCGGCCACCTATTG |
| 231-T-R | AATAACGCCACCGGTACCCTGAATCGGTTCGCCAACAAATGCTGCCACTT |
| 231-W-F | GAACCGATTCAGGGTTGGGGTGGCGTTATTGTTCCGCCGGCCACCTATTG |
| 231-W-R | AATAACGCCACCCCAACCCTGAATCGGTTCGCCAACAAATGCTGCCACTT |
| 231-Y-F | GAACCGATTCAGGGTTATGGTGGCGTTATTGTTCCGCCGGCCACCTATTG |
| 231-Y-R | AATAACGCCACCATAACCCTGAATCGGTTCGCCAACAAATGCTGCCACTT |
| 231-V-F | GAACCGATTCAGGGTGTGGGTGGCGTTATTGTTCCGCCGGCCACCTATTG |
| 231-V-R | AATAACGCCACCCACACCCTGAATCGGTTCGCCAACAAATGCTGCCACTT |
| 231-F-F | GAACCGATTCAGGGTTTTGGTGGCGTTATTGTTCCGCCGGCCACCTATTG |
| 231-F-R | AATAACGCCACCAAAACCCTGAATCGGTTCGCCAACAAATGCTGCCACTT |
| 231-test-F | CGCAAGGAATGGTGCATGCA |
| 231-test-R | TTTGGTTTTCAGCCGGATCT |
| zwf-F | TACCATGGGCAGCAGCCATCACCATCATCACCACgcggtaacgcaaacagccca |
| zwf-R | CGCCGAGCTCGAATTCGGATCCTGGCTttactcaaactcattccaggaacgaccat |
| zwf-GJ-F | agtaaAGCCAGGATCCGAATTCGAG |
| zwf-GJ-R | GTGATGATGGTGATGGCTGCTGCCCATGGTATATCTC |
| pAZ-test-F | GTGAGCGGATAACAATTCCCCTG |
| pAZ-test-R | GATTATGCGGCCGTGTACAA |
| pos5-F | ATGGGCAGCAGCCATCACCATCATCACCACagtacgttggattcacattccctaaag |
| pos5-R | CGAGCTCGAATTCGGATCCTGGCTttaatcattatcagtctgtctcttggtcagcc |
| pos5-GJ-F | tgattaaAGCCAGGATCCGAATTCGAG |
| pos5-GJ-R | ccaacgtactGTGGTGATGATGGTGATGGCTGCTGCCCATGGTATATCT |

**References**

1. Kunjapur AM, Tarasova Y, Prather KL. Synthesis and accumulation of aromatic aldehydes in an engineered strain of Escherichia coli. J Am Chem Soc. 2014;136(33):11644-54.
