# Supplementary figures and images for: Construction and yield optimization of a cinnamylamine biosynthesis route in Escherichia coli
Source: Biotechnol Biofuels Bioprod. 2022 Sep 29;15:100. doi: 10.1186/s13068-022-02199-7 (PMC9524069; doi:10.1186/s13068-022-02199-7)

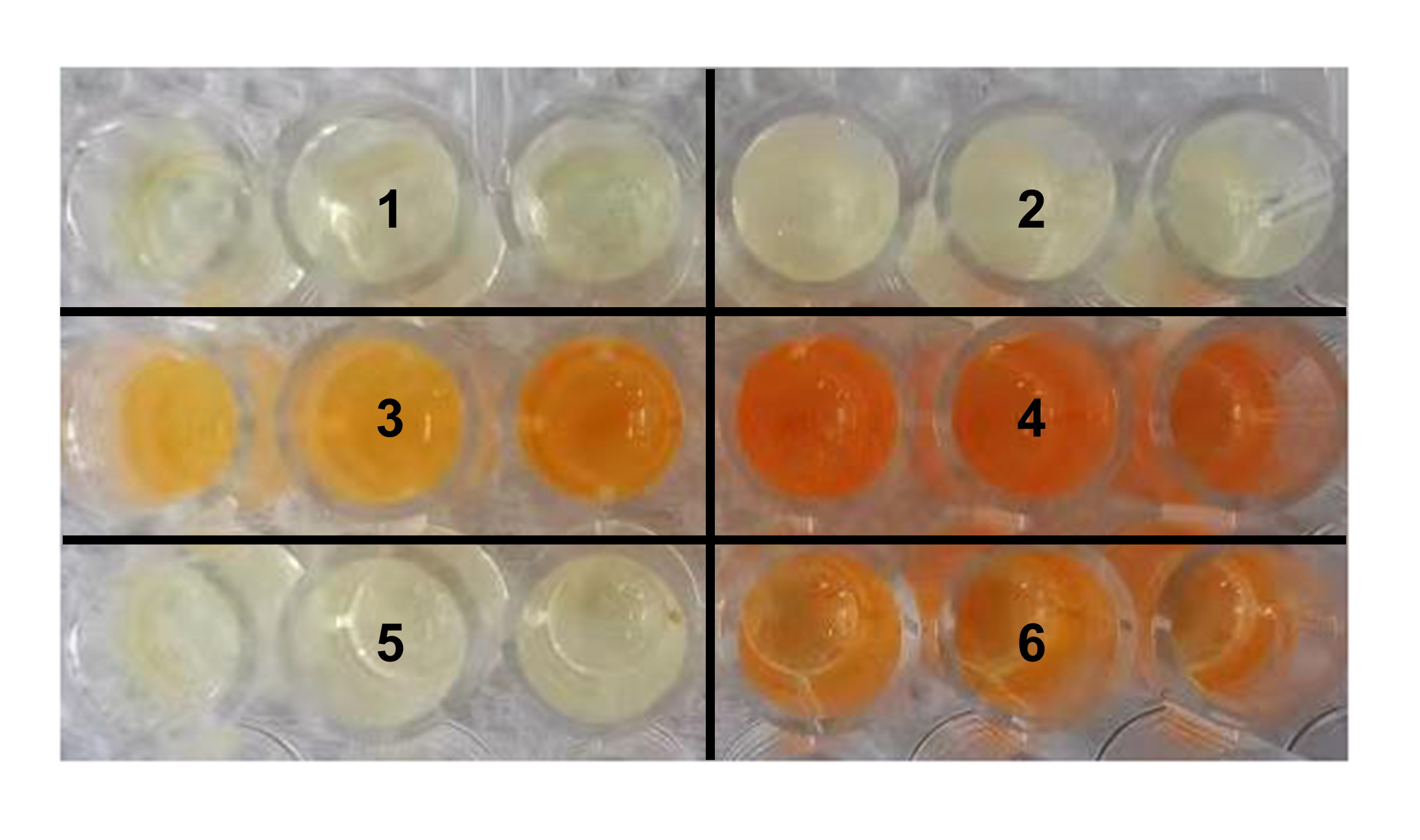

Supplement: Supplementary file 4 — Additional file 4: Figure S1. Validation of ω-transaminase activity based on color reaction. 1: Control 2: Empty vector plasmid strain breaking solution 3: Breaking solution of strain expressing He-ωTA protein 4: Breaking solution of strain expressing Cv-ωTA protein 5: Purified He-ωTA protein solution 6: Purified Cv-ωTA protein solution. [file 13068_2022_2199_MOESM4_ESM.tif]

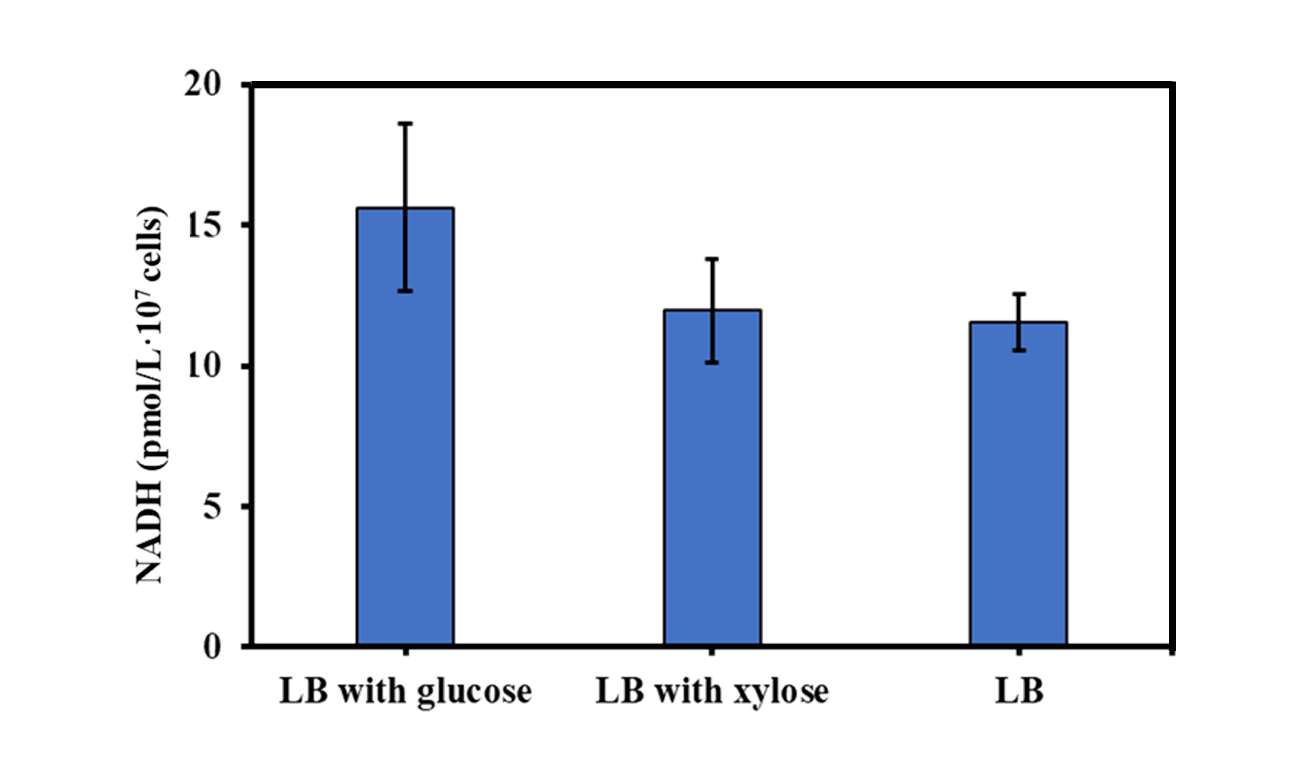

Supplement: Supplementary file 5 — Additional file 5: Figure S2. Intracellular NADH content of MER-oE strains under different medium conditions. Data represent mean ± S.D. (error bars) from three independent experiments. [file 13068_2022_2199_MOESM5_ESM.tif]

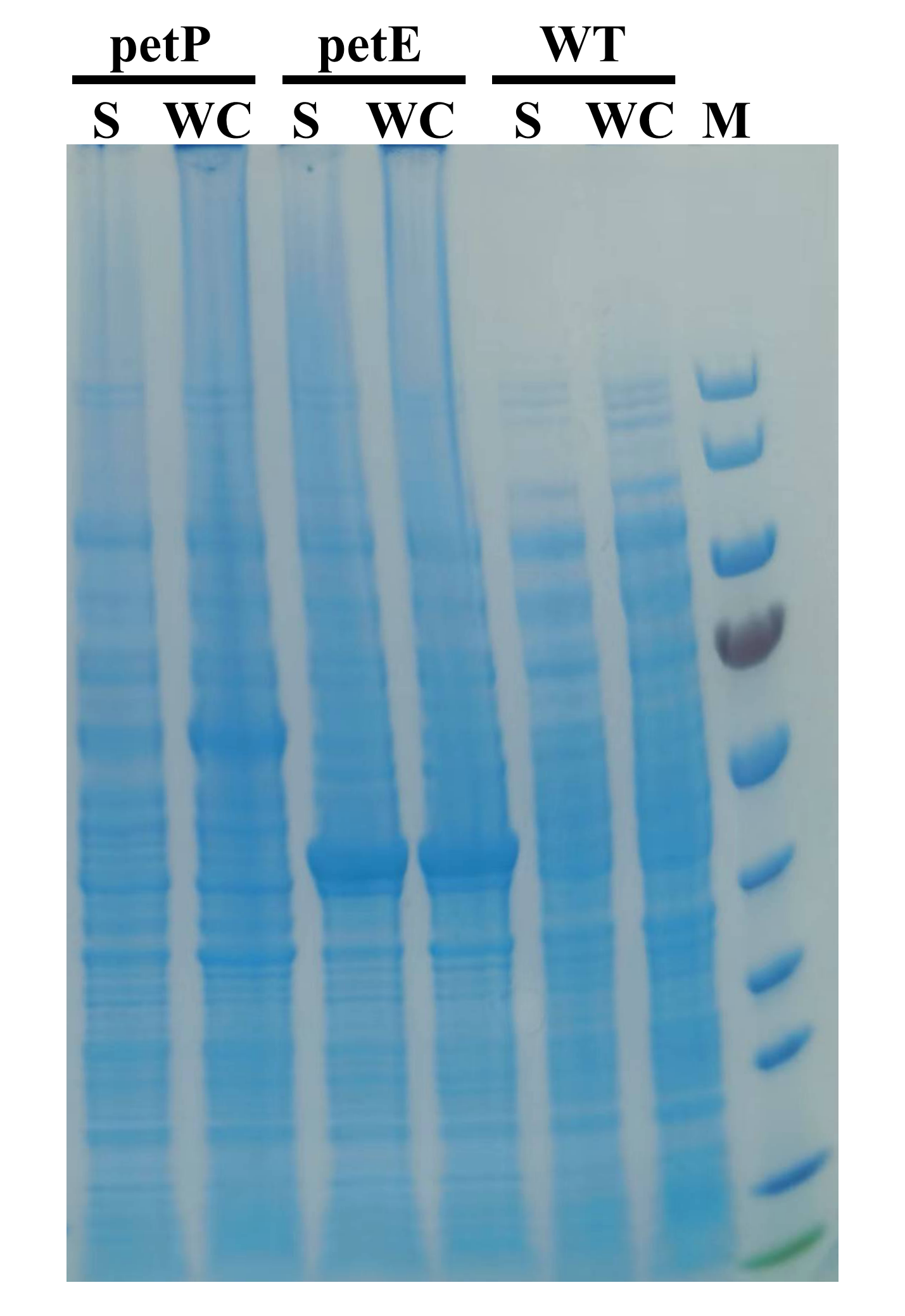

Supplement: Supplementary file 6 — Additional file 6: Figure S3. The petP and petE plasmids expression in E. coil by SDS-PAGE analysis of whole cell (WC), supernatant (S), and protein marker (M). [file 13068_2022_2199_MOESM6_ESM.tif]

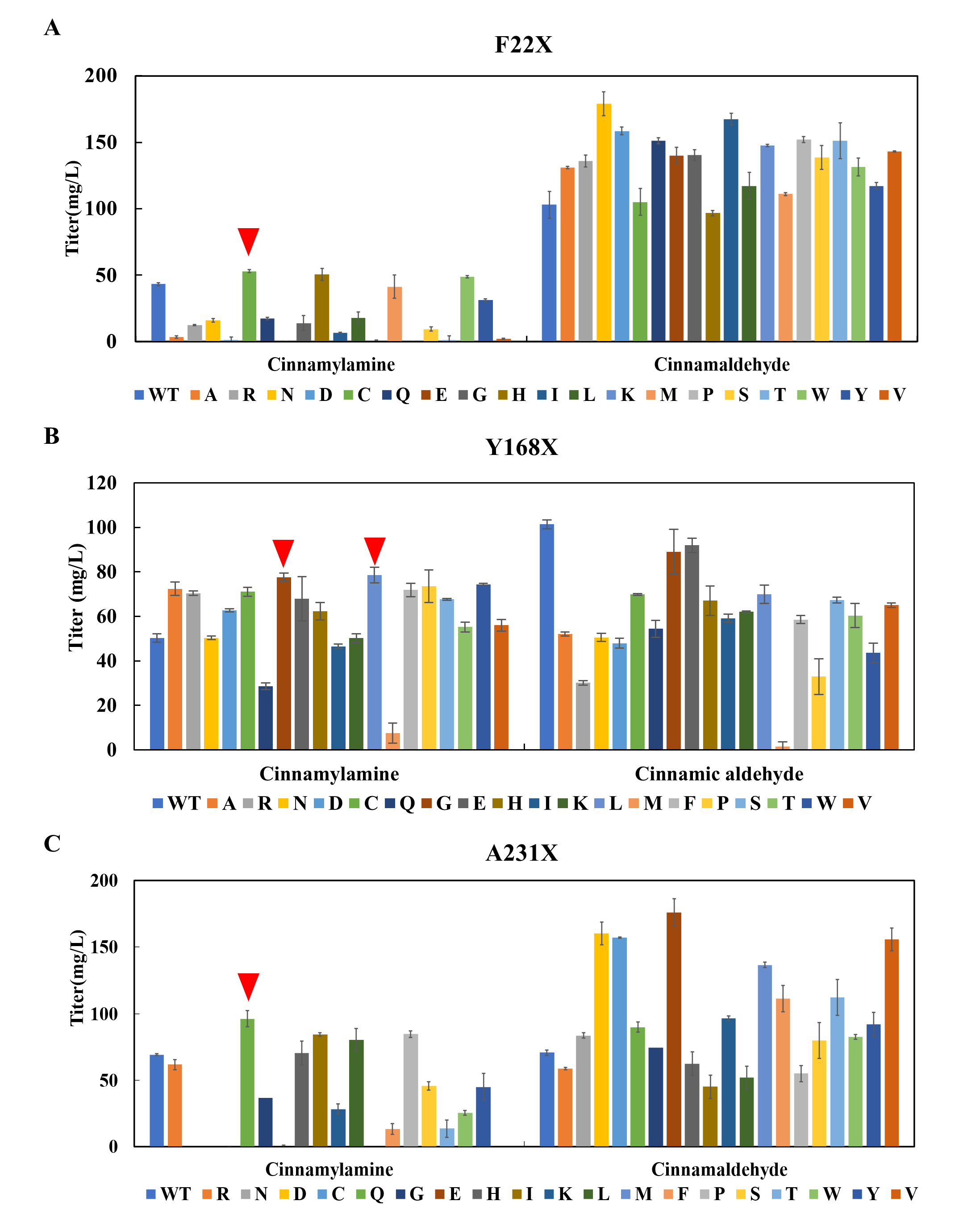

Supplement: Supplementary file 7 — Additional file 7: Figure S4. Yields of saturated mutants of three amino acid residues of Cv-ωTA converting cinnamaldehyde to cinnamylamine. (A) The production of cinnamylamine in saturated mutants of at the Phe22 site after adding cinnamaldehyde. (B) The production of cinnamylamine in saturated mutants of at the Tyr168 site after adding cinnamaldehyde. (C) The production of cinnamylamine in saturated mutants of at the Ala231 site after adding cinnamaldehyde. The mutant with the highest cinnamylamine production among the saturated mutants at each site is marked with a red inverted triangle. "X" represents any amino acid. Data represent mean ± S.D. (error bars) from three independent experiments. [file 13068_2022_2199_MOESM7_ESM.tif]
